# Suggested spontaneous resolution of possible paediatric hydrosalpinx: a case report with discussion

**DOI:** 10.1007/s10397-015-0925-1

**Published:** 2015-12-09

**Authors:** Zainab Kazmi, Sujata Gupta, Michael Dobson

**Affiliations:** University of Manchester School of Medicine, Stopford Building, Oxford Road, Manchester, M13 9PT UK; Women’s Health Directorate, Royal Preston Hospital, Sharoe Green Lane North, Preston, Lancashire PR2 9HT UK; Department of Radiology, Royal Preston Hospital, Sharoe Green Lane North, Preston, Lancashire PR2 9HT UK

**Keywords:** Paediatric hydrosalpinx, Abdominal tenderness, Gynaecological sonographer

## Abstract

Hydrosalpinx is a rare cause of abdominal pain in paediatric patients, though cases are documented in the literature. Its aetiology differs considerably from traditional hydrosalpinx due to ascending sexually transmitted infection. Hydrosalpinx can resent mimicking an acute abdomen or can be asymptomatic. Management of paediatric hydrosalpinx varies but often involves surgical removal of the affected tube. A 12-year-old girl presented with left-sided acute abdominal pain setting within 24 h. Initial ultrasound scan suggested presence of hydrosalpinx. Post-discharge follow-up appointment with a consultant paediatric gynaecologist demonstrated no symptomology, but repeated scan by another sonographer showed continued presence of possible hydrosalpinx, which had since grown. Later, MRI was performed to confirm site of the lesion. However, MRI revealed no tubal masses, suggesting spontaneously resolved hydrosalpinx. Consultant-administered ultrasound scan confirm no tubal abnormalities. Our case suggests spontaneous resolution in possible paediatric hydrosalpinx. Our recommendation is for conservative management of asymptomatic paediatric and adolescent hydrosalpinges, with emergency surgery offered if symptoms indicative of tubal or adnexal torsion.

## Case report

A 12-year-old nulliparous girl presented to paediatric admissions unit with acute onset abdominal pain centralized to the left iliac fossa over the past 12 h, leading to one episode of sudden collapse. She reported no associated bowel or urinary symptoms. The patient had reached menarche aged 12 and had regular menstrual cycles. She denied any prior sexual activity, and her last menstrual period has occurred 1 week prior. She had no prior surgical or medical history of note. Social and family history was unremarkable.

On examination, abdominal tenderness was noted with no associated guarding or peritonism. Midstream urine and beta-hCG levels were tested and recorded, with normal findings. The patient was admitted for observation. However, the pain resolved within 24 h and no further symptoms were reported following resolution. Admission notes suggested initial working diagnosis was of an ovarian cyst. The patient was discharged and scheduled for a follow-up review and ultrasound scan in 2 weeks.

**Fig. 1:**
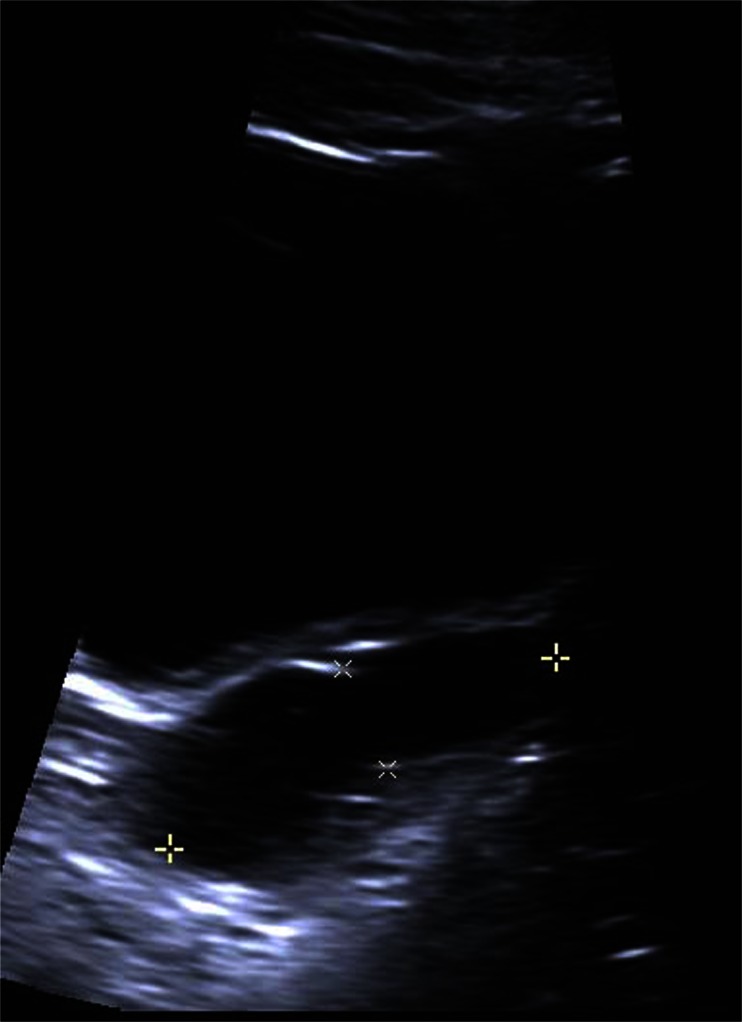
Initial ultrasound image (shows pathology)

On the initial follow-up 2 weeks after discharge, an outpatient ultrasound scan was performed by an experienced gynaecological sonographer (Fig. 1). The sonographer reported an elongated fluid-filled structure within the left adnexa adjacent to the uterus measuring 5 × 1.3 cm ‘consistent with hydrosalpinx’. There were no other adnexal pathology noted, and there was no evidence of free fluid.

**Fig. 2:**
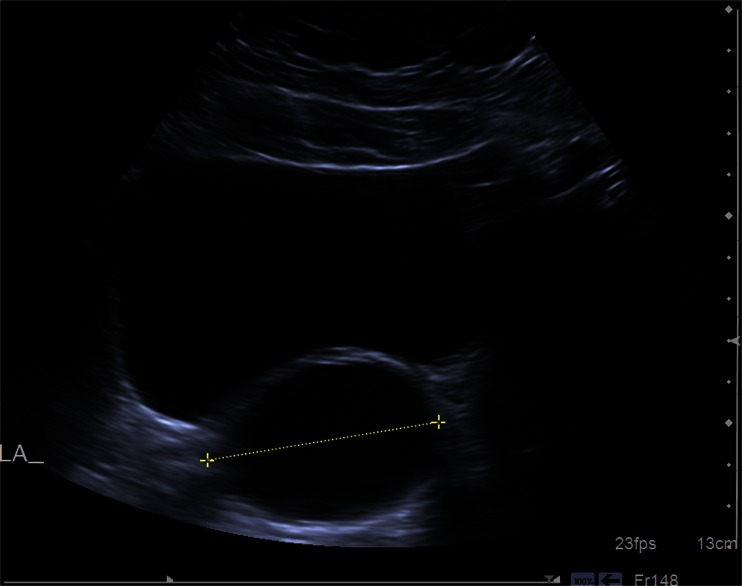
Follow-up ultrasound image (shows pathology)

Due to missed appointments, the next follow-up scan was performed 6.5 months later by a different experienced gynaecological sonographer (Fig. 2). This sonographer reported the presence of an 8.2 × 3 × 5.6 cm fluid-filled tubular structure in the left adnexa. She remarked also that the ultrasound appearances of an elongated cyst are suggestive of hydrosalpinx. No other adnexal pathology was seen, with no evidence of free fluid in the pelvis.

**Fig. 3:**
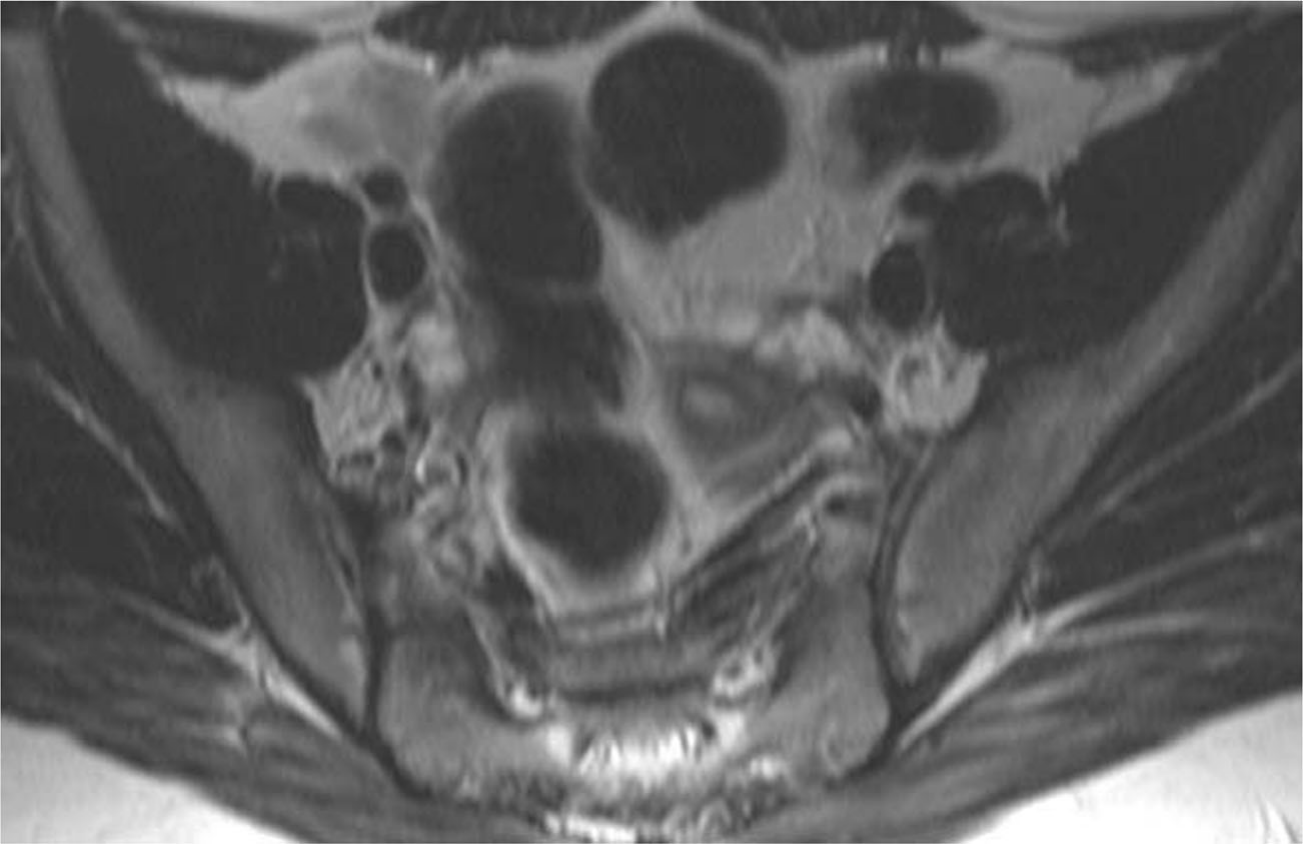
MRI image (no pathology)

With the continued presence of what appeared to be a possible hydrosalpinx, a laparoscopy and salpingectomy were scheduled. Prior to the operation, a consultant radiologist-administered MRI of the pelvis was performed with the aim of confirming the site of the hydrosalpinx (Fig. 3). However, this MRI demonstrated no evidence of hydrosalpinx or any pelvic visceral abnormalities.

Due to these new imaging findings, a decision was made to review the patient using the original imaging modality: ultrasound. A pelvic ultrasound, using a trans-abdominal approach and performed by a consultant radiologist, demonstrated no evidence of a pelvic cyst or mass or any free fluid.

As there were no further troubling symptoms and no abnormalities reported on two separate imaging modalities, no treatment was indicated. The patient was reviewed one final time in follow-up clinic. The patient was discharged from gynaecology outpatient follow-up clinic. There has been no recurrence in symptomology reported as of time of writing.

## Discussion

### Aetiology

Adult hydrosalpinx is traditionally associated with pelvic inflammatory disease due to an ascending sexually transmitted infection such as *Chlamydia trachomatis* [1]. However, the aetiology in paediatrics differs considerably. In paediatric patients, resultant peritubal adhesions from past inflammation or surgery can lead to pelvic venous congestion, which contribute to distal tubal occlusion disclosure [2]. Pelvic venous congestion may be caused by adnexal masses, usually either inflammatory or functional, like the corpus luteum [2]. Neoplastic adnexal masses, however, are very rare in this age range [2]. Overall, the pathogenesis of hydrosalpinx remains poorly defined in children [3]. In this case, there was no positive history for any of the above causes. Furthermore, the patient became and remained asymptomatic after admission.

Without clear pre-existing tubal or ovarian pathology, paediatric hydrosalpinx is likely due to increased fallopian tube mobility on the ligamentum latum [1]. As well, the perimenarcheal (early puberty) phase can be associated with higher follicle-stimulating hormone (FSH) levels due to the stimulated hypothalamic-pituitary-gonadal axis, which leads to new activation and increased mobility of ovarian and tubal function [1, 2]. This can cause a previously asymptomatic hydrosalpinx to become newly symptomatic [1].

New onset symptomology of previously asymptomatic hydrosalpinx can also be due to abnormal tubal peristalsis activity or inherent congenital tubal abnormalities [2]. These anatomical abnormalities include abnormal mesosalpinx length, spiral course of the salpinx and distal occlusion [2].

### Presentation

Amongst paediatric patients, hydrosalpinx appears to be most common in the perimenarcheal phase [2], possibly due to the factors listed above.

Clinical onset of fallopian tube disease is highly variable. There is often associated pain, but occasionally, no pain may be noted [2]. Where the hydrosalpinx is symptomatic, it is often associated with nonspecific signs of infection [3]. Unfortunately, the nonspecific symptomology can contribute to difficulty making a diagnosis. Moreover, often, ongoing symptomology is only present in cases of continuing infection [4].

### Investigations

Imaging is paramount in diagnosing hydrosalpinx. In children with acute or subacute-onset pelvic pain, trans-abdominal ultrasound is the preferred initial imaging modality [5]. Ultrasonography is cost-effective and very safe to use [5]. It can also give instantaneous answers and is useful as an initial imaging modality [6].

Ultrasonography of hydrosalpinx can easily demonstrate dilatation at the ampulla [2] or an elongated cystic pathology in the fallopian tubes [6] that is separated from and leading to the ovary [2]. Colour Doppler ultrasonography can provide evidence of hydrosalpinx by demonstrating increased vascularity and decreased resistance index (RI) when the cause is neoplastic or infective [1].

Magnetic resonance imaging is also useful to differentiate hydrosalpinx from other similar presentations [2, 5]. Fluid contents become apparent on MRI with low T1 and high T2 signals [2]. A different appearance is seen in haematosalpinx and pyosalpinx, based on the density of cystic content [2].

Imaging is also useful in allowing us to determine whether dilatation is acute or chronic. More acute conditions are demonstrated by the ‘cogwheel sign’, which shows subtle, linear projections protruding into the lumen [2]. Chronic conditions are shown as ‘beads on a string’, with small mural foci and flattened mucosal folds [2].

Furthermore, we can review potential complications using imaging. On both ultrasonography and MRI, a characteristic whirlpool sign is classically noted where tubal torsion is present [2]. This is critical as the earlier torsion is diagnosed and treated, the much higher the probability that the fallopian tube can be salvaged [1].

However, it is not possible to make a definite diagnosis without diagnostic surgery, thought this is obviously invasive. The benefits of surgical investigation, like laparoscopy, include possibility of simultaneous treatment if an abnormality is detected [6].

### Future advances in imaging

There remain some drawbacks of using ultrasonography [7], including the possibility that ultrasonography alone is not always accurate. 3D ultrasound is a promising new imaging modality that avoids the common misdiagnosis of hydrosalpinx that often occurs with 2D sonography [8]. Presently, diagnostic surgery may be the most accurate imaging modality. A retrospective surgical study revealed that, for hydrosalpinx, there is a 100 % association between laparoscopic and final histological findings [9]. However, invasive approaches should be avoided wherever possible. Advances in imaging may pave the way for a more thorough pre-operative assessment [10].

### Complications

Hydrosalpinx is a known cause of fallopian tubal torsion, which can have an adverse impact on fertility due to possible tubal and ovarian necrosis. Moreover, torsion itself can result in acute abdominal pain, nausea and vomiting. Torsion is three times more frequent on the right tube than left, likely due to the cushioning effect of the sigmoid colon on the left tube [11]. Isolated tubal torsion is rare, with its incidence being reported as one in 1.5 million women [11]. Torsion of a normal tube has been previously reported, though incidence is dramatically higher where there is pre-existing tubal or ovarian pathology [4].

Hydrosalpinx itself can be painful with the enlargement of a mass in the abdomen. This can change with the waxing and waning of hydrosalpinx with reabsorption of hydrosalpinx and subsequent re-accumulation [12].

### Management

Fallopian tube pathologies, including both ovarian cysts and hydrosalpinx, indicate surgical intervention when presenting as an acute abdomen [2]. A low threshold is required for surgical intervention as the associated symptomology for torsion is nonspecific [13].

The types of surgeries offered vary. Salpingectomy refers to the surgical removal of a Fallopian tube, and is often preferred over other surgeries where the tube and ovary are preserved, as those operations have a higher risk of future ectopic pregnancies. While salpingectomy is most often indicated for ectopic pregnancies, it is also used to manage tubal damage, as in hydrosalpinx.

Historically, salpingectomies were completed using a laparotomy approach. With technological advances, a laparoscopic approach is increasingly favoured as it avoids the larger laparotomy incision, leading to less post-operative pain and quicker discharge [5].

Bilateral salpingectomy is a cause of female sterility and should be avoided. Hydrosalpinx resection has previously been recommended where there is no functional ipsilateral ovary [5].

Other surgical procedures which may be considered include salpingostomy, salpingopexy and detorsion. Salpingostomy is a tubal corrective surgery and eliminates the need for tubal loss. However, it is also associated with a significantly increased rate of ectopic pregnancy post-operatively [14]. Salpingopexy refers to the fixation of the tube via suturing to the posterior broad ligament of the uterus or to the lateral pelvic wall with the intent of preventing future tubal or adnexal torsion [15]. However, this runs the risk of changing the normal anatomy of the pelvic organs [16]. Detorsion is relevant where the hydrosalpinx has caused the adnexa or the fallopian tube to tort. The untwisting of the tube can reduce the risk of ischaemic damage as it allows the potential for revascularization [17]. Another more conservative measure that may be implemented in the future is the aspiration of the hydrosalpinx, guided by ultrasound, through the abdominal wall [11]. This procedure can avoid an incision altogether.

In paediatrics, all cases requiring surgery must be considered carefully, with the decision to operate being weighed against the use of an invasive management approach in asymptomatic minors. Furthermore, often, paediatric operations can be inherently more challenging, with variable anatomy and aetiologies. The decision to operate in paediatric hydrosalpinx presents its own unique set of challenges. In managing paediatric hydrosalpinx, there is a fine line between under-treatment and over-treatment. In under-treatment, there is a risk of tubal or ovarian torsion, recurrence, tubal pregnancy and infertility [2]. With over-treatment, one runs the risk of subjecting a child to unnecessary surgery [2].

### Prognosis

Hydrosalpinx often adversely affects future fertility. The presence of this structure can cause inflammation of the fallopian tubes, and free uterine fluid can impact implantation [18]. The presence of this structure can also block movement of the ovum into the uterine cavity [19].

There are reports of resolution of hydrosalpinx in adult patients in the literature. The findings from this case presentation are quite novel because a spontaneous resolution of this condition in paediatrics has never been clearly reported in the literature. However, in all cases of paediatric hydrosalpinx, a decision to operate is made quickly after presentation and diagnosis. Perhaps, in prior cases, due to the speed in operating, there was insufficient time for a spontaneous resolution.

### Recommendations

Given that this case report suggests potential for spontaneous resolution, our proposed management for conservative management is as follows:

1. Utilize a watch-and-wait approach for asymptomatic hydrosalpinx. Ensure that patients and parents/guardians are educated on the condition and all potential risks.
2. Routine ultrasound scanning to assess changes in hydrosalpinx size/shape/location.
3. If the hydrosalpinx is symptomatic and suggestive of possible tubal/ovarian torsion, emergency surgery should be offered, with detorsion or salpingectomy depending on potential for revascularization.

## Conclusion

We have presented this case report of a 12-year-old girl with what appears to be spontaneous resolution of possible hydrosalpinx not requiring any medical or surgical management. Paediatric hydrosalpinx is often treated with surgery, particularly salpingectomy, in order to prevent future risk of ovarian torsion and thus to preserve fertility. However, salpingectomy can be associated with reduced fertility as well. We hope to propose a case for a watch-and-wait approach without any intervention for asymptomatic possible hydrosalpinx in order to minimize any unnecessary treatment in a paediatric patient.
